# O6.7. COMMON NEUROANATOMICAL ABNORMALITIES IN FIRST EPISODE PSYCHOSIS ACROSS SEVERAL INDEPENDENT SAMPLES

**DOI:** 10.1093/schbul/sby015.228

**Published:** 2018-04-01

**Authors:** Sandra Vieira, Cristina Scarpazza, Benedicto Crespo-Facorro, Diana Tordesillas-Gutierrez, Victor Ortiz-García de la Foz, Esther Setien-Suero, Floor Scheepers, Qiyong Gong, Tiago Reis Marques, Robin Murray, Anthony David, Paola Dazzan, Andrea Mechelli

**Affiliations:** 1 Institute of Psychiatry, Psychology and Neurosciences, King’s College London; 2 Marqués de Valdecilla University Hospital, Instituto de Investigación Valdecilla, CIBERSAM; 3 University Medical Center Utrecht; 4 School of Public Administration, Sichuan University

## Abstract

**Background:**

Structural abnormalities in first episode psychosis (FEP) tend to be subtle and widespread. Most studies investigating structural abnormalities in this clinical population have used small samples, and therefore may be under-powered. In addition, most studies have examined participants at a single research site, and therefore the results may be specific to the local sample investigated. Consequently, findings from existing studies have often been heterogeneous. This study aimed to overcome these issues by testing for neuroanatomical abnormalities in individuals with FEP relative to healthy controls that are expressed consistently across five independent datasets.

**Methods:**

Structural Magnetic Resonance Imaging data were acquired from a total of 572 patients with FEP and 502 age and gender comparable healthy controls (HC) at five sites - London (UK), Utrecht (Netherlands), Chengdu (China) and two independent sites at Santander (Spain). Voxel-based morphometry (VBM) as implemented in Statistical Parametric Mapping software (SPM12) was used to investigate differences in gray matter volume (GMV) between the two groups. The statistical analysis was carried out using an analysis of variance (ANCOVA), with diagnostic group and scanning site as factors, and age and gender as covariates of no interest. Neuroanatomical alterations in patients with FEP relative to HC common to the five datasets were identified by comparing the total FEP group against the total HC group, and then using the inclusive masking option (at p<0.05 uncorrected) to identify those regions that survived the comparison between FEP and HC within each dataset. Individual clinical scores from each site were normalised and then used to examine their association with GMV in the total sample. Statistical inferences were made at p<0.05 after family-wise error correction for multiple comparisons.

**Results:**

Relative to HC, FEP showed a widespread pattern GMV reduction in fronto-temporal regions bilaterally, including the gyrus rectus, orbitofrontal, temporal, fusiform, precentral and lingual gyri, anterior cingulate and insula as well as in the parietal lobe in the precuneus gyrus. The largest GMV reduction was found in the left gyrus rectus which is part of the inferior frontal lobe. Negative correlations were found between this region and positive symptoms severity (r=-.2, p<.001) and duration of illness (r=-.1, p<.012), but not with negative symptoms (r=.0, p=.991). Patients also showed GMV increases in the temporal gyrus bilaterally, left inferior frontal gyrus and right cerebellum relative to HC.

**Discussion:**

This study identified a common pattern of fronto-temporal-parietal reductions in five independent FEP samples; in addition, some of these reductions were more pronounced in patients with more severe positive symptoms and longer duration of illness. This pattern of results suggests the presence of symptom- and stage-dependent neuroanatomical alternations in FEP that are expressed above and beyond site-related differences in recruitment criteria and scanning parameters.

